# Recurrence of Chronic Subdural Hematoma Is Independent of ABO Blood Type: A Retrospective Cohort Study

**DOI:** 10.3389/fneur.2022.833958

**Published:** 2022-05-20

**Authors:** Yunwei Ou, Xiaofan Yu, Liang Wu, Dong Zhang, Weiming Liu

**Affiliations:** ^1^Department of Neurosurgery, Beijing Tiantan Hospital, Capital Medical University, Beijing, China; ^2^Beijing Neurosurgical Institute, Capital Medical University, Beijing, China; ^3^China National Clinical Research Center for Neurological Diseases, Beijing, China; ^4^Beijing Advanced Innovation Center for Big Data-Based Precision Medicine, Beihang University, Beijing, China; ^5^Neurological Center, People's Hospital of Ningxia Hui Autonomous Region, Yinchuan, China

**Keywords:** chronic subdural hematoma, ABO blood type, clinical features, recurrence, burr-hole craniostomy

## Abstract

**Objective:**

A previous study with a limited number of patients has shown that blood type A was a risk factor in the recurrence of CSDH. The primary objective of this study was to investigate the association between the recurrence of CSDH and ABO blood type based on a larger sample size.

**Methods:**

The authors reviewed in-patients with symptomatic CSDH from August 2011 to August 2021. Hospitalization information and data on long-term outcomes and recurrence among these patients were gathered. For all clinical variables, numbers (percentages) and mean ± standard deviations were used for categorical and continuous variables, respectively. For intergroup comparisons, the χ2 test or one-way ANOVA was carried out. Multivariable logistic regression analyses were performed to identify the association between CSDH recurrence and blood types.

**Results:**

We included 1,556 inpatients in this study. The recurrence rate of CSDH showed no differences among different blood types. In the multivariable logistic regression analyses, ABO blood type (A: OR, 1.064; 95% CI, 0.467–2.851, *p* = 0.793; B: OR, 0.682, 95% CI, 0.315–1.269, *p* = 0.164; AB: OR, 0.537, 95% CI, 0.426–1.861, *p* = 0.357) was not a significantly independent predictor of CSDH recurrence.

**Conclusions:**

Our study demonstrated that ABO blood type was not a risk factor in the recurrence of CSDH. Thus, we should not pay too much attention to ABO blood type in terms of CSDH recurrence.

## Introduction

Chronic subdural hematoma (CSDH) is a neurosurgical disorder that is characterized as a progressive blood aggregation between the arachnoid mater and the dura mater (1, 2). The number of the elderly patients diagnosed with CSDH is increasing due to an aging society (3). Although the prognosis of patients with CSDH after surgery is relatively good, a major concern in CSDH is that the lesion often recurs after surgery (10–20%) (4–7). There are several risk factors in the higher recurrence of CSDH, involving bilateral hematomas, large hematoma diameter, postoperative residual hematoma volume, and types of hematoma (8, 9). In addition, our previous research has indicated that diabetes mellitus, use of urokinase, and complications could also be significant predictors for the recurrence of CSDH (10). The pathogenic mechanisms of CSDH recurrence are complicated and considered to be related to the inflammatory processes (11, 12). Recent studies have illustrated that blood group, also known as “blood type,” may be a factor in the inflammatory progression (13).

The human ABO blood group antigens consist of intricate glycan structures on glycolipids and glycoproteins (14). These antigens are displayed on the surface of various cells and tissues, including platelets, blood cells, vascular endothelium, and neuronal cells (15, 16). ABO blood type associates with diverse pathological conditions, such as carcinoma, infection, and vascular disorders (17). Notably, increasing evidence has revealed that hemostasis and thrombosis might be associated with the ABO blood type (18, 19). A retrospective clinical study has reported that blood type A remarkably related to the higher recurrence of CSDH concerning on the population of Japan (20). However, the size of the subjects of this retrospective study was limited. Here, this study was based on a larger sample size to retrospectively investigate the association between the recurrence of CSDH and ABO blood type.

## Methods

We identified 1,630 inpatients with symptomatic CSDH at Beijing Tiantan Hospital, Capital Medical University from August 2011 to August 2021. Symptomatic CSDH was defined as a clear diagnosis of CSDH by computed tomography (CT) scan, and the inpatients had neurological symptoms, such as headache, dizziness, limb weakness, and dysphasia. ABO blood typing was not performed on 74 inpatients. The remaining 1,556 inpatients whose ABO blood type was determined were included in this study. However, only three of 1,556 patients were Rh-negative blood. Therefore, we did not further analyze the Rh blood type to avoid data bias. An exhaustive drainage strategy in burr-hole craniostomy was performed in all the enrolled inpatients, which has been reported in our previous study (10). Age, gender, and medical history were collected from each patient. If the patients had been taking anti-thrombotic agents within 7 days before admission,they should stop taking anti-thrombotic medications for 7 days due to the surgical operation after admission in the hospital. It was recommended that the patients should be tested by preoperative coagulation tests, involving activated partial thromboplastin time (APTT), prothrombin time (PT), thrombin time (TT), and the international normalized ratio (INR). As previously described, the hematomas were divided into seven types (containing A type: hypodense; B type: isodense; C type: hyperdense; D type: laminar; E type: separated; F type: gradation; and G type: trabecular) based on the density of subdural collections (21). Then, we carefully recorded the postoperative complications, such as heart attack, fever, and seizure. Two neurosurgical residents were contacted with the enrolled patients about their situations at 6 months after discharge. The modified Rankin scale (mRS) was performed to evaluate the outcomes of the patients at 6 months. The scores of mRS were represented as 0–3, marking the good outcome and were represented as 4–6, marking the poor outcome. The recurrence was defined as appearance of symptoms and signs attributable to the ipsilateral hematoma, reoccurring within 6 months after the first surgery. This study was approved by the Institutional Review Board of Beijing Tiantan Hospital, Capital Medical University (Ethical inspection No. KY2020-094-02). Written informed consent was obtained from all the enrolled patients.

### Statistical Analyses

We performed statistical analyses through using IBMSPSS Statistics (version 22.0; IBM Corp.). For all clinical variables, numbers (percentages) and mean ± standard deviations were used for categorical and continuous variables, respectively. Prior to statistical analyses, relevant data of the patients were checked for normality. And non-normally distributed data were analyzed by using non-parametric tests. For intergroup comparisons, the χ2 test or one-way ANOVA was carried out. The association between CSDH recurrence and blood types was determined by multivariable logistic regression analyses. All tests were 2-sided, and *p* < 0.05 was considered significant.

## Results

ABO blood typing was not examined in 74 inpatients, and we included 1,556 inpatients in this study. As presented in Table 1, the mean ages of the patients with blood types A, B, AB, and O were 62.1 ± 17.3 years, 65.2 ± 15.3 years, 65.6 ± 13.8 years, and 62.6 ± 16.8 years, respectively. There were differences in age between the patients of different blood types (*p* = 0.045). Further analysis found that the patients with blood type B were older than those with blood type A (*p* = 0.041) and O (*p* = 0.039) (Table 2). In terms of gender, the proportions of men in the four groups were 83.1% (blood type A), 81.8% (blood type B), 84.4% (blood type AB), and 80.2% (blood type O) (*p* = 0.603). As for comorbidities, we found differences in the proportion of diabetes among the patients of different blood types (*p* = 0.013). The percentage of diabetes was highest in the patients with blood type AB (29.9%). Moreover, as indicated in Table 2, the differences in the proportion of diabetes between blood type AB and the other three blood types were statistically significant (blood type A vs. blood type AB, *p* = 0.002; blood type B vs. blood type AB, *p* = 0.010; blood type AB vs. blood type O, *p* = 0.006). However, there were no significant differences in the other three comorbidities (hypertension, cardiac diseases, and brain infarction) between different blood types. Regarding clinical symptoms (headache, dizziness, limb weakness, and dysphasia), no differences were shown among the four groups. The proportions of coagulopathy among the patients of different blood types also occurred statistically significant (*p* = 0.035). We discovered that a higher percentage of the patients with blood type B suffered coagulopathy compared to blood type A through further statistical analysis (*p* = 0.005). However, the percentage of the patients taking antithrombotic drugs did not show differences between the various blood groups (*p* = 0.898). History of taking antithrombotic medications (*p* = 0.898), bilateral hematoma (*p* = 0.915), and diameter of hematoma (*p* = 0.689) also showed no differences among the patients with different blood types. Types of hematoma did not significantly differ between the patients of different blood types (*p* = 0.073).

**Table 1:** Preoperative clinical features of 1,556 patients with CSDH.

		**Blood Type**				
**Variable**	**Total**	**A**	**B**	**AB**	**O**	***p***
No. of patients	1,556	390 (25.1)	554 (35.6)	147 (9.4)	465 (29.9)	
Age in yrs	63.3 ± 15.9	62.1 ± 17.3	65.2 ± 15.3	65.6 ± 13.8	62.6 ± 16.8	0.045
Men	1,274 (81.9)	324 (83.1)	453 (81.8)	124 (84.4)	373 (80.2)	0.603
Hypertension	542 (34.8)	131 (33.6)	191 (34.5)	59 (40.1)	161 (34.6)	0.547
Diabetes mellitus	312 (20.1)	68 (17.4)	111 (20.0)	44 (29.9)	89 (19.1)	0.013
Cardiac diseases	42 (2.7)	10 (2.6)	12 (2.2)	8 (5.4)	12 (2.6)	0.182
Brain infarction	180 (11.6)	43 (11.0)	66 (11.9)	16 (10.9)	55 (11.8)	0.965
Headache	902 (58.0)	212 (54.4)	335 (60.5)	85 (57.8)	270 (58.1)	0.319
Dizziness	437 (28.1)	103 (26.4)	148 (26.7)	44 (29.9)	142 (30.5)	0.442
Limb weakness	851 (54.7)	203 (52.1)	317 (57.2)	88 (59.9)	243 (52.3)	0.156
Dysphasia	147 (9.4)	34 (8.7)	55 (9.9)	11 (7.5)	47 (10.1)	0.731
Disturbance of consciousness	67 (4.3)	20 (5.1)	24 (4.3)	5 (3.4)	18 (3.9)	0.741
Coagulopathy	533 (34.3)	114 (29.2)	211 (38.1)	46 (31.3)	162 (34.8)	0.035
Antithrombotic drugs	172 (11.1)	41 (10.5)	64 (11.6)	18 (12.2)	49 (10.5)	0.898
Bilateral hematoma	411 (26.4)	106 (27.2)	141 (25.5)	38 (25.9)	126 (27.1)	0.915
Maximum transverse diameter of hematoma in cm	2.2 ± 0.8	2.2 ± 0.5	2.3 ± 0.8	2.1 ± 0.8	2.1 ± 0.6	0.689
Types of hematoma						0.073
A	260 (16.7)	66 (16.9)	96 (17.3)	21 (14.3)	77 (16.6)	
B	431 (27.7)	107 (27.4)	162 (29.2)	38 (25.9)	124 (26.7)	
C	322 (20.7)	85 (21.8)	114 (20.6)	34 (23.1)	89 (19.1)	
D	119 (7.6)	35 (9.0)	41 (7.4)	9 (6.1)	34 (7.3)	
E	68 (4.4)	16 (4.1)	25 (4.5)	3 (2.0)	24 (5.2)	
F	69 (4.2)	21 (5.4)	22 (4.0)	11 (7.5)	15 (3.2)	
G	287 (18.4)	54 (13.8)	88 (15.9)	36 (24.5)	109 (23.4)	

**Table 2:** Comparisons of age, diabetes, and coagulopathy between different blood types.

**Blood type**	***p* (Age)**	***p* (Diabetes)**	***p* (Coagulopathy)**
A vs. B	0.041	0.316	0.005
A vs. AB	0.053	0.002	0.641
A vs. O	0.898	0.522	0.081
B vs. AB	0.587	0.010	0.129
B vs. O	0.039	0.719	0.284
AB vs. O	0.064	0.006	0.429

As shown in Table 3, there were no differences in reduction of hematoma cavity (*p* = 0.336) and duration of the drainage catheter (*p* = 0.461) between different blood types. Furthermore, symptoms disappeared or alleviated (*p* = 0.752) and complications (*p* = 0.519) also showed no differences among the patients with different blood types. In terms of urokinase, there were no differences in use of urokinase (*p* = 0.145) and frequency of urokinase used (*p* = 0.679) between different blood types. Significantly, the recurrence rate of CSDH (*p* = 0.414) and outcomes (*p* = 0.762) showed no differences among different blood types. In the multivariable logistic regression analyses, blood types (ABO blood typing as A, B, AB, and O) (A: OR, 1.064, 95% CI, 0.467–2.851, *p* = 0.793 B: OR, 0.682, 95% CI, 0.315–1.269, *p* = 0.164; AB: OR, 0.537, 95% CI, 0.426–1.861, *p* = 0.357) were not significantly independent predictors of CSDH recurrence (shown in Table 4).

**Table 3:** Postoperative clinical features of 1,556 patients with CSDH.

		**Blood Type**				
**Variable**	**Total**	**A**	**B**	**AB**	**O**	***p***
No. of patients	1,556	390 (25.1)	554 (35.6)	147 (9.4)	465 (29.9)	
Reduction of hematoma cavity (%)	62.0 ± 21.5	62.4 ± 22.9	61.3 ± 21.6	62.1 ± 20.8	61.8 ± 22.4	0.336
Symptom disappeared or alleviated (day)	2.5 ± 2.1	2.5 ± 2.2	2.6 ± 2.2	2.6 ± 2.1	2.5 ± 2.1	0.752
Duration of drainage catheter (day)	3.2 ± 1.9	3.3 ± 1.6	3.1 ± 1.4	3.4 ± 1.7	3.2 ± 2.1	0.461
Use of urokinase	809 (52.0)	197 (50.5)	302 (54.5)	65 (44.2)	245 (52.7)	0.145
Frequency of urokinase used	1.8 ± 1.1	1.9 ± 1.1	1.7 ± 1.2	1.6 ± 1.2	1.8 ± 1.1	0.679
Complications	103 (6.6)	24 (6.2)	36 (6.5)	14 (9.5)	29 (6.2)	0.519
Outcomes (mRS)						0.762
0–3	1,521 (97.8)	381 (97.7)	544 (98.2)	144 (98.0)	452 (97.2)	
4–6	35 (2.2)	9 (2.3)	10 (1.8)	3 (2.0)	13 (2.8)	
Recurrence	65 (4.2)	19 (4.9)	17 (3.1)	6 (4.1)	23 (4.9)	0.414

**Table 4:** Multivariable logistic regression analysis of the relationship between CSDH recurrence and blood types.

**Factor**	**Crude model**		***p***
	**OR**	**95% CI**	
Blood type			
O	Reference		
A	1.064	0.467–2.851	0.793
B	0.682	0.315–1.269	0.164
AB	0.537	0.426–1.861	0.357

## Discussion

This study was based on a larger sample to explore the relationship between the recurrence of CSDH and blood types. Our results suggested that blood types were not remarkably associated with the higher recurrence of CSDH.

The ABO antigens were originally found on the surface of red blood cells, so it is also called the ABO blood group antigens. Importantly, growing evidence has shown that the ABO blood group antigens were expressed on the surface of a variety of cells and tissues as well, including platelets, blood cells, vascular endothelium, and sensory neurons (15, 16). Thus, it is understandable that ABO blood type is related to various conditions. A survey of the elderly over the age of 100 from Japan indicated that blood type B may be a sign of longevity (22). Interestingly, patients with CSDH, with blood type B, were older than those with blood types A and O in our study. CSDH is a disease that predominates in the elderly; the longer life expectancy of the elderly with blood type B may help explain the finding of the relationship between blood type B and age in this study. Furthermore, ABO blood type has been recently reported to be associated with various diseases (13, 18). A study evaluating the relationship between ABO blood type and diabetes demonstrated that people with blood type B were at highest risk of developing diabetes followed by people with blood type AB (23). Furthermore, we found that the percentage of diabetes was highest in patients with CSDH with blood type AB by analyzing our data. Therefore, this point may also be consistent with previous research on the association between blood types and diabetes to some extent. Besides, hemorrhage and hemostasis might also be associated with the ABO blood type. We discovered that a higher percentage of the patients with blood type B suffered coagulopathy compared to blood type A.

The current research on the onset and progression of CSDH is still in the initial stage. Vascular endothelial growth factor (VEGF) was observed in the subdural fluid and outer membrane of hematoma (24). Additionally, a previous study has proposed that patients with enhanced expression of VEGF and basic fibroblast growth factor in outer membrane had a higher risk of the recurrence of CSDH (25). Interestingly, ABO blood type has been recently reported to be related to vascular endothelial growth factor receptor 2 (VEGFR2) and VEGFR3. Furthermore, the average levels of VEGFR2 and VEGFR3 were lowest in blood type A compared to the other three blood types (13). Therefore, blood types may influence the onset and development of CSDH by affecting the biological effects of VEGF. For instance, a recent retrospective study has suggested that blood type A was significantly associated with the recurrence of CSDH (20). However, our study did not find a relationship between blood types and recurrence. So, whether ABO blood type can influence the development and recurrence of CSDH by affecting VEGF needs to be further verified.

Many clinical studies have investigated the independent risk factors in the recurrence of CSDH. A retrospective cohort study indicated that postoperative hematoma volume and types of hematoma were predictors of CSDH recurrence after surgery (9). Moreover, another single-center retrospective study showed that bilateral hematoma and increased hematoma diameter were independent risk factors in the recurrence of CSDH (8). Our previous data based on a larger sample size also confirmed that the characteristic of hematoma (bilateral hematoma and postoperative hematoma volume) was a risk factor in the recurrence of CSDH. Apart from that, our results demonstrated that use of urokinase and postoperative complications were independent risk factors in the recurrence of CSDH (10). The clinical use of urokinase may indicate that complex hematoma components are more likely to cause the recurrence of CSDH. Thus, we believe that the type of hematoma can affect the progression and recurrence of CSDH. A recent study has supported that the patients with gradation-density hematoma were an independent predictor for CSDH recurrence (26). What is more, the association between characteristics of hematoma and ABO blood type has also been reported in several pieces of literature. A retrospective study that enrolled patients of acute subdural hematoma found that blood type A was related to the degree of the midline shift and postoperative seizures (19). Another retrospective study based on a larger sample size demonstrated that blood type O was related to the higher risk of hematoma expansion in patients with intracerebral hemorrhage (27). The above two studies have reached different conclusions. Nevertheless, both of the above two studies discussed the progression of acute hematoma. Only one article that focused on the association between the recurrence of CSDH and ABO blood type was found by consulting relevant literature. Hirai et al. included 320 patients of CSDH and concluded that blood type A was highly associated with the recurrence of CSDH (20). Our study did not find that blood types were related to the recurrence of CSDH based on a larger size of patients. Moreover, the recurrence rate of CSDH in our institute was much lower than the recurrence rate reported by Hirai et al. We speculated that the exhaustive drainage strategy may be the main reason for this result (10). Thus, it is inappropriate to pay more attention to blood type for the recurrence of CSDH. The local inflammation, characteristics of hematoma, and surgical strategies are noteworthy.

There are several limitations to this study. Firstly, the study was a single-center retrospective study; the causal association between the recurrence of CSDH and blood type is tentative. Secondly, the number of patients was relatively sufficient, while all the patients were Chinese; these findings may not be generalized to non-Asian people. Thirdly, the ABO blood type was not performed on all the patients; despite that all the enrolled patients met the diagnosis of symptomatic CSDH, selection bias was unavoidable.

## Conclusions

In sum, this study concluded that ABO blood type was not a risk factor in the recurrence of CSDH. Thus, we should not pay too much attention to ABO blood type in terms of CSDH recurrence.

## Data Availability Statement

The raw data supporting the conclusions of this article will be made available by the authors, without undue reservation.

## Ethics Statement

The studies involving human participants were reviewed and approved by IRB of Beijing Tiantan Hospital, Capital Medical University. The patients/participants provided their written informed consent to participate in this study. Written informed consent was obtained from the individual(s) for the publication of any potentially identifiable images or data included in this article.

## Author Contributions

DZ and WL designed the research. YO and XY collected data, drafted the manuscript, took the responsibility for the integrity of the data, and the accuracy of the data analysis. LW supervised the project. All the authors contributed to the article and approved the submitted version.

## Funding

This work was supported by the National Natural Science Foundation of China (81502150), the Institute of Artificial Intelligence, Hefei Comprehensive National Science Center (21KT013), the Capital's Funds for Health Improvement and Research (2020-2-2045), the Capital Health Research and Development of Special (2022-2-2047), Beijing Advanced Innovation Center for Big Data-Based Precision Medicine, Beihang University, Beijing, China, National Natural Science Foundation of China (81930048), Capital Characteristic Clinical Application Project (Z181100001718196), and National Key Technology Research and Development Program of the Ministry of Science and Technology of China (2014BAI04B01, 2015BAI12B04, and 2013BAI09B03).

## Conflict of Interest

The authors declare that the research was conducted in the absence of any commercial or financial relationships that could be construed as a potential conflict of interest.

## Publisher's Note

All claims expressed in this article are solely those of the authors and do not necessarily represent those of their affiliated organizations, or those of the publisher, the editors and the reviewers. Any product that may be evaluated in this article, or claim that may be made by its manufacturer, is not guaranteed or endorsed by the publisher.

## Abbreviations

CSDH: chronic subdural hematoma
mRS: modified Rankin Scale
CT: computed tomography
INR: international normalized ratio
APTT: activated partial thromboplastin time
PT: prothrombin time
TT: thrombin time
VEGF: vascular endothelial growth factor
VEGFR: vascular endothelial growth factor receptor.
